# Drug Delivery Challenges in Brain Disorders across the Blood–Brain Barrier: Novel Methods and Future Considerations for Improved Therapy

**DOI:** 10.3390/biomedicines9121834

**Published:** 2021-12-04

**Authors:** Aneesha Achar, Rosemary Myers, Chaitali Ghosh

**Affiliations:** 1 Cerebrovascular Research, Department of Biomedical Engineering, Lerner Research Institute, Cleveland Clinic, Cleveland, OH 44195, USA; ava28@case.edu (A.A.); myersr4@ccf.org (R.M.); 2 Department of Biomedical Engineering and Molecular Medicine, Cleveland Clinic Lerner College of Medicine of Case Western Reserve University, Cleveland, OH 44195, USA

**Keywords:** blood–brain barrier, drug bioavailability, current routes, drug regulatory mechanism, CNS disorders

## Abstract

Due to the physiological and structural properties of the blood–brain barrier (BBB), the delivery of drugs to the brain poses a unique challenge in patients with central nervous system (CNS) disorders. Several strategies have been investigated to circumvent the barrier for CNS therapeutics such as in epilepsy, stroke, brain cancer and traumatic brain injury. In this review, we summarize current and novel routes of drug interventions, discuss pharmacokinetics and pharmacodynamics at the neurovascular interface, and propose additional factors that may influence drug delivery. At present, both technological and mechanistic tools are devised to assist in overcoming the BBB for more efficient and improved drug bioavailability in the treatment of clinically devastating brain disorders.

## 1. Introduction

**Figure 1:**
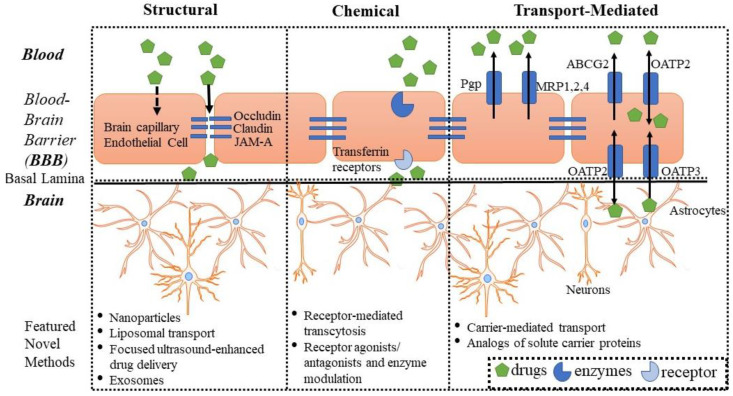
Schematic representation of potential targets and selective novel strategies for drug delivery to the brain across the blood-brain barrier (BBB).

The blood–brain barrier (BBB) is a semipermeable interface in the central nervous system (CNS) that exhibits critical properties responsible for regulating CNS homeostasis. Although the BBB is present in most brain regions, blood vessels in the circumventricular organs (including area postrema, median eminence, neurohypophysis, pineal gland, subfornical organs, and lamina terminalis) have fenestrations that allow the diffusion of molecules from the blood across the vessel wall [1]. On the other hand, the blood–CSF barrier of the choroid plexus is unique in that it has both tight junction and adherens junction proteins and is more permeable [1]. The physiological barrier itself is composed primarily of endothelial cells, which form the thin blood vessel wall. Glial cells, namely astrocytes, tightly surround the endothelial cells of the capillaries, with neurons in close proximity, and regulate BBB function [2,3]. Neurovascular communication is vital for a healthy brain and proper drug distribution [4,5]. Other cell types such as pericytes, oligodendrocytes, smooth muscles cells, etc. in the periphery also play an important role in maintaining brain homeostasis, thereby retaining healthy BBB function. Additionally, tight junction proteins, including occludin, claudins, and junctional adhesion molecule-A, prevent the paracellular movement of large serum proteins and electrolytes between endothelial cells [6,7]. Therefore, the BBB forms a strict structural boundary between the blood vessel lumen and the abluminal brain [8]. In addition to the physical barrier of the BBB, brain endothelial cells exhibit enzymatic activity capable of degrading certain molecules prior to crossing the BBB [9]. Brain endothelial cells also express nutrient transporters (e.g., solute carrier proteins) and important transport proteins that control the passage of several xenobiotics and drugs (Figure 1), including P-glycoprotein/MDR1 (Pgp), multidrug resistance-associated protein 1 (MRPs), breast cancer-resistant protein (BCRP), and the organic anion transporters (OATs), which may efflux substances back into the blood circulation and prevent the accumulation of molecules in the brain [9,10]. However, the OATs have been localized to almost all barrier epithelia of the body, as well as the endothelium, and have demonstrated roles in the regulated transcellular movement of numerous small organic anionic molecules across these epithelial barriers and between body fluid compartments (e.g., blood–central nervous system, blood–urine, intestine–blood, blood–bile, blood–placenta, and others). Although prototypical members of this transporter family are capable of the bidirectional movement of substrates, most of the OATs are generally viewed as influx transporters, facilitating the movement of organic anions into the epithelial and endothelial cells [11].

For these reasons the neuroprotective functions of the diseased BBB evidently present a challenge to drug delivery to the brain, especially in brain disorders where efflux transporters are overactive [12,13,14]. As a direct result, drugs may be unable to reach target tissue or achieve sufficient concentration in the brain, a challenge that has been reported in a variety of CNS disorders to date [15]. Due to its relevance in the medical field and pharmaceutical industry, drug delivery has thus become a prominent focus of research within the past two decades. Recently, several studies and research endeavors have demonstrated innovative methods to circumvent the BBB in drug delivery [16,17,18], which is further discussed in this review.

## 2. Common Methods to Treat Selective Central Nervous System Disorders

In this section, we provide a short description of treatments currently utilized in medical practice, focusing on the limitations of each treatment method as they relate to the BBB. Novel methods of drug delivery recently proposed or utilized in selected CNS disorders are summarized in Table 1.

### 2.1. Epilepsy

Epilepsy is a complex neurological disorder characterized by frequent, unprovoked seizures resulting from hypersynchronous neuronal discharge in the brain [40,41,42]. The treatment of epilepsy features a wide range of anti-seizure drugs (ASDs) that primarily target specific ion channels and neurotransmitters, thereby controlling abnormal electrical activity in the epileptic brain [43,44,45,46]. Although ASDs may manage the majority of epilepsy cases, the long-term systemic use of these drugs can cause adverse side effects [47]. The disease pathogenesis and long-term treatment regimen might play a role in adverse side effects [48].

According to the International League Against Epilepsy, approximately one-third of patients with epilepsy are diagnosed with refractory (pharmacoresistant) epilepsy, in which frequent seizures persist despite treatment with two or more anti-seizure medications or other therapies [40,49,50,51,52]. Although the exact cause of drug resistance in epilepsy is still unclear, many studies have suggested BBB involvement during epileptogenesis and seizure onset [53,54,55]. Moreover, a number of the cytochrome P450 (CYP) drug metabolizing enzymes involved in drug metabolism along with drug transporters have been found to be overexpressed and overactive in human epileptic brain regions, particularly at the BBB [56,57]; the gene variant hypothesis of these targets associated with ASD pharmacokinetics and pharmacodynamics cannot be ruled out. Specifically, variations in genes that encode enzymes that metabolize ASDs or ion channels and neurotransmitter receptors targeted by ASDs can potentially affect ASD response. These results suggest that the local biotransformation of anti-seizure medication or dysfunctional drug transport mechanisms at the BBB may compromise the therapeutic index in the target brain region. Additionally, efflux transport proteins such as Pgp and BCRP were also found to be significantly upregulated in human epileptic brain regions versus non-epileptic regions, suggesting an increased efflux of anti-seizure medications under the diseased condition in accordance with the transporter hypothesis of refractory epilepsy [58,59]. More recently, the relevance of upstream molecular regulators/transcription factors such as glucocorticoid receptors (GR) and pregnane X receptors (PXR) and their control of drug metabolizing enzymes and drug transporters has been noted in the human epileptic brain, especially at BBB endothelial cells [60,61,62].

In addition to the gene variant and transporter hypotheses, several other explanations of pharmacoresistant epilepsy exist. The neural network hypothesis suggests that post-seizure neural network remodeling results in the suppression of the endogenous anti-seizure system, thereby preventing anti-seizure medications from reaching their respective targets [59,63]. Conversely, the intrinsic severity and target hypotheses propose that pharmacoresistance is inherent to disease severity or the decreased drug sensitivity of neurotransmitter receptors and voltage-gated ion channels to anti-seizure medications, respectively [59,64]. As evident by multiple hypotheses, there is no single mechanism of drug resistance; rather, a combination of biological and pharmacokinetic properties may contribute to drug delivery challenges in this specific disease condition.

### 2.2. Stroke

The main types of stroke include ischemic stroke, which accounts for approximately 80% of cases, and hemorrhagic stroke, which accounts for the remaining minority [65,66]. Ischemic stroke may result from a thrombotic event, in which blood vessel dysfunction obstructs blood flow to the brain, or an embolic event, in which debris or clots from another origin site become dislodged in the vessel, thereby blocking blood flow [67,68]. An “abrupt neurological outburst” follows, including necrosis, leukocyte infiltration, oxidative stress, and BBB impairment [68]. Stroke medications include a tissue plasminogen activator for clot breakdown and anticoagulants, antiplatelets, and antihyperintensive medications aimed at reducing common risk factors. However, due to rapid mechanisms of cell death, time is a significant limiting factor of stroke treatment [69]. For example, a tissue plasminogen activator must be administered within 3–4.5 h of ischemic insult [70,71]. Even then, studies have reported that the intravenous administration of a tissue plasminogen activator in proximal vessel occlusion failed to restore blood flow in 60% of patients [72,73]. The bioavailability of stroke drugs is also limited by decreased blood flow to the penumbra [69].

As mentioned previously, BBB disruption has been implicated in ischemic stroke [74,75]. One possible mechanism of BBB disruption reported in hypoxia/ischemia is mediated by matrix metalloproteinase (MMP) activation. More specifically, oxygen and ATP loss induce hypoxia inducible factor-1α (HIF-1α), which activates pro-membrane-type 1 metalloproteinase-MMP (proMMP-2) via the convertase furin. Simultaneously, the inflammatory cytokines tumor necrosis factor- α (TNF-α) and interleukin-1β (IL-1β) activate MMP-3, which further activates MMP-9 with the aid of free radicals. Upon activation, MMP-2 and MMP-9 degrade endothelial basil lamina and tight junctions, subsequently disrupting BBB integrity and aggravating cerebral edema [74]. Aside from MMP, the pleiotropic cytokine macrophage migration inhibitory factor (MIF) also contributes to BBB dysfunction in ischemic stroke [25,76]. MIF binds to receptors expressed on endothelial cells, causing the induction of endothelial cell autophagy and increased vascular permeability, and promotes the production of pro-inflammatory cytokines including TNFα, IL-1β and IL-6. The administration of MIF in a rat model of ischemic stroke has been found to disrupt tight junction expression and increase BBB permeability, bilateral infarct volume, and stroke injury [25].

The disruption of BBB integrity following ischemic injury is a multifaceted issue. Although it may be tempting to associate compromised BBB and improved drug delivery based on the structural properties of the BBB, past studies of other central nervous system disorders have shown that a “leaky” BBB does not necessarily indicate increased drug delivery [77]. Moreover, BBB disruption in ischemic stroke is predictive of a negative outcome, as leukocyte, T-cell, and other immune cell invasion into the CNS results in post-stroke inflammation and exacerbated brain injury [75,78]. Finally, the increased brain concentration of tissue plasminogen activator, the most common stroke medication, may result in plasmin activation and further hemorrhagic complications [79]. Therefore, anti-stroke methods that may preserve BBB structure or function in this pathological condition, such as targeting MIF, and potentially enhance the drug delivery window would be highly beneficial (see Table 1).

### 2.3. Brain Cancer

Brain cancer is a generalized term to describe unregulated cell growth involving mutation, the activation of oncogenes and the deactivation of tumor suppressor genes, cell cycle dysregulation, and the inhibition of apoptotic mechanisms that causes tumor growth in the brain [80,81]. Common types of brain tumors include oligodendrogliomas, meningioma, and diffuse astrocytic tumors (astrocytoma, anaplastic astrocytomas, and glioblastomas) in adults, and pilocyticastrocytomas, ependymomas, and medulloblastomas in children [82]. Among this list, glioblastomas are particularly aggressive malignant brain tumors involving cell necrosis [83,84]. Treatment methods aimed at both managing and eliminating malignant brain tumors include surgical resection, radiation therapy, and chemotherapy. Chemotherapy features the administration of anti-cancer drugs, each of which interfere with DNA, RNA, and protein synthesis or cancer cell function via a unique mechanism of action [85]. For example, anthracycyclinedoxorubicin inhibits DNA and RNA synthesis. Additionally, the antibody bevacizumab specifically targets vascular endothelial growth factor. Nitrosoureascarmustine and lomustine inhibit DNA repair pathways [85]. These examples demonstrate the variety of mechanisms of anti-cancer drug function. However, as these drug regimens may affect both cancerous and healthy cells, unintended effects of chemotherapy have been well reported, including nausea and vomiting, fatigue, decreased appetite, changes in taste, hair loss, dry mouth, and constipation [86,87].

Even though multiple anti-cancer drugs thus far have been developed and passed FDA approval, the number applicable for brain cancer and cure is limited. Chemotherapeutic agents approved for brain tumors include everolimus, bevacizumab, carmustine, naxitamab-gqgk, and temozolomide [88]. The severe limitation is because the vast majority of anti-cancer drugs are unable to cross the BBB [89]. This is primarily the result of efflux transporter activity at the brain vasculature, including Pgp, MRPs, BCRP, and the influx transporter and/or bi-directional transporter such as OATs, as discussed previously [90,91,92].

Tumor growth during metastasis in the brain may partially disrupt BBB function, whereas the “blood–brain–tumor barrier” constitutes another obstacle compromising the vascular integrity [93,94]. Structural differences in the BBB and blood–brain–tumor barrier include irregular pericyte distribution and astrocytic processes, increased immune cell presence, and decreased endothelial cell junctional protein expression, all resulting in compromised permeability properties in comparison to the normal BBB. However, the blood–tumor barrier also exacerbates the presence and overactivity of efflux transport proteins and thereby continues to pose a hurdle for anti-cancer drug delivery [94]. Characteristics of the tumor itself may also influence BBB/blood–tumor barrier permeability to anti-cancer drugs, as observed for example in the childhood brain tumor medulloblastoma [95]. More specifically, the mutant signaling molecule β-catenin was found to induce hemorrhagic vasculature in the mouse wingless (WNT) subtype, and as a direct result, BBB permeability is increased. Therefore, WNT-medulloblastoma is highly responsive to chemotherapy, leading to better outcomes in vivo. Conversely, without the induction of hemorrhagic vasculature in the Sonic Hedgehog subtype of mice, the BBB remains undisturbed. Chemotherapy may, therefore, be ineffective toward Sonic Hedgehog-medulloblastoma, causing drug resistance in this subtype [95].

### 2.4. Traumatic Brain Injury

Traumatic brain injury (TBI) is an acquired brain injury resulting in a wide range of neurological deficits, from mild, temporary alterations of consciousness to comatose and death, depending on the severity of primary and secondary injuries [96]. Primary injury is the direct effect of physical trauma to the brain, potentially consisting of intracranial and extracranial hemorrhage, tissue destruction, and axonal shearing [97]. Secondary injury, however, occurs minutes to years after the primary injury and often involves the progression of possible BBB dysfunction via multiple mechanisms.

More specifically, TBI may disrupt the integrity of microvessels in the BBB, causing the activation of the coagulation cascade. Coagulation could result in focal ischemia near the site of trauma, thereby reducing blood flow to these brain regions. Compromised BBB permits thrombin, albumin, fibrinogen, and other blood-borne factors into the brain, which may cause the activation and proliferation of microglia and the production of pro-inflammatory factors [98]. TBI-induced brain trauma may also interfere with neurovascular unit communication, causing astrocytes and microglial to produce molecules including transforming growth factor-β (TGF-β), reactive oxygen species, vascular endothelial growth factor (VEGF), MMPs, and glutamate. The elevation of such molecules destroys or downregulates tight junction expression, resulting in decreased BBB integrity and potential downstream changes, including cerebral edema [98].

The treatment of TBI varies significantly based on injury severity and type of injury. For mild injuries or sub-concussion, treatment consists of rest and pain medication. However, severe injuries may require immediate emergency care. To prevent further neurological damage, the treatment of secondary injuries features surgery to address blood clots, stop bleeding, repair skull fractures, and relieve cranial pressure; medication, including anti-seizure drugs, coma-inducing drugs, and diuretics; and rehabilitation. However, as in the case of stroke, time may be a limiting factor of TBI treatment. In clinical care settings, the “golden hour” refers to the first 60 min following primary injury in which medical treatment is critical to patient outcomes, a concept which has been debated in scientific literature. However, a retrospective study on human patients with head injuries scored 3 or higher on the abbreviated injury scale found a significant association between patient mortality and each additional minute of hospital arrival time [99]. Results also indicate an observed survival benefit for patients arriving within 120 min of primary injury, suggesting that time remains an important factor in patient outcomes, but the optimal time frame may extend beyond the “golden hour” [99]. As a result, there exists a need for rather fast-acting novel methods to restore proper BBB functioning and prevent further neurological damage in TBI.

## 3. Methods to Combat Structural, Chemical, and Transport-Mediated Challenges of Drug Delivery across the BBB

As presented in Figure 1 and discussed in detail in the Introduction, BBB properties that disrupt sufficient drug delivery to the brain may be classified into structural, chemical, and transport-mediated challenges. However, certain novel drug delivery methods function by circumventing or targeting these specific challenges.

### 3.1. Structural Challenges

#### 3.1.1. Nanoparticles

Nanoparticles are solid colloidal particles of matter between 1 and 100 nanometers in size [100] and vary significantly in composition, featuring liposomes, solid lipid nanoparticles, non-polymeric micelles, lipoplex, dendrimers, polymeric nanoparticles, polymeric micelles, nanotubes, silica nanoparticles, quantum dots, gold nanoparticles, and magnetic nanoparticles [101]. It is hypothesized that the utilization of such nanoparticles as drug vehicles may enhance BBB targeting and crossing. More specifically, upon loading drugs into nanoparticles, the chemical composition of the drug no longer obstructs BBB crossing. Rather, the physicochemical and biomimetic properties of nanoparticles determine BBB crossing [101]. The proper evaluation of the chemical composition of the nanoparticle itself is critical for determining its toxicity to minimize adverse effects when brought to clinic.

Evidence thus far supports the hypothesis of nanoparticle-assisted drug delivery. In one recent study, fluorescently labeled glucose-coated gold nanoparticles were loaded with the anti-seizure medication lacosamide (LCM-GNP). In the experiment, gold nanoparticles and LCM-GNP were intravenously administered in Wistar albino rats with temporal lobe epilepsy and control rats [23]. Results indicate that LCM-GNP significantly decreased the amplitude and frequency of EEG waves and positive-going activity measured 24 h post-administration in epileptic rats. Epileptic rats administered LCM-GNP also exhibited higher hippocampal concentrations of nanoparticles, perhaps suggesting the need for dosage reevaluations in drug-resistant epilepsy, and lower glutathione levels [23], a potential indication of decreased oxidative stress.

In another recent study, chitosan–lecithin nanoparticles loaded with the anti-seizure medication phenytoin were intranasally administered in conscious mice [24]. The drug delivery of phenytoin is particularly challenging due to the drug’s low aqueous solubility and tendency to crystallize, causing slow absorption and post-injection pain. Additionally, phenytoin is both highly protein-bound and classified as a Pgp substrate, resulting in low brain bioavailability. However, the study found that the intranasal administration of phenytoin-loaded chitosan–lecithin nanoparticles in BALBc mice resulted in a rapid accumulation of phenytoin in the mouse brain after 5 and 60 min, along with sustained concentrations 72 h after administration [24].

Although these two pieces of evidence involve nanoparticle-assisted drug delivery in epilepsy, nanoparticles have been applied to other neurological disorders as well, such as brain cancer. For example, Cornell prime dots, fluorescent core–shell silica nanoparticles, were functionalized with avintegrin-binding peptides (cRGD) and labeled with PET labels [30]. Functionalized dots were then administered to a mouse model of RCAS/tv-a glioblastoma. The immunofluorescence of murine tissue revealed high cRGD ligand densities and a corresponding high cellular binding of cRGD-functionalized dots. Additionally, the distribution of the tumor-specific Cy5 signal resembled the distribution of the intravenous injection of FITC-labeled dextran (comparable to C’ dot size), showing colocalization in disrupted BBB regions at an early time point; however, cRGD-C’ dots were also able to distribute beyond disrupted BBB regions. Finally, the manipulation of incubation temperatures also suggested the uptake of cRGD-C’ dots was receptor-mediated, as decreased incubation temperatures resulted in the decreased quantity of Cy5 cells [30], demonstrating that nanoparticles not only address structural BBB challenges, but may utilize receptor-mediated mechanisms as well.

Although the structural barrier of the BBB poses many challenges to therapy, in diseases like TBI, the barrier is often significantly damaged, allowing unwanted molecules from the blood to enter the brain, leading to inflammation (see above section on Traumatic Brain Injury). This transient BBB damage can be utilized for the delivery of therapeutics via nanoparticles [102]. An issue with this method to deliver drugs using TBI BBB pathophysiology is the heterogeneity of the condition, the scale of the damage, and that the time to endogenous BBB repair is variable between patients. Rather than use the BBB damage to deliver the nanoparticles, a recent study in a TBI mouse model found success in delivering Tau-siRNA using nanoparticles coated with polysorbate 80 (PS 80) via the lipoprotein receptor [34]. Although not yet tested in humans, this therapy seems promising for the delivery of therapeutics across the BBB in patients with TBI.

#### 3.1.2. Liposomal Transport

Liposomes are small (0.025 to 2.5 μm), spherical, artificial vesicles composed of phospholipids and cholesterol, thereby exhibiting both hydrophobic and hydrophilic properties [103]. Beyond this basic structure, liposomal properties may differ depending upon saturation, bilayer components, charge, size, preparation method, and surface modifications [103]. Similar to the rationale behind nanoparticle-assisted drug delivery, liposomal transport involves the utilization of liposomes as a drug delivery vehicle.

Recently, liposomal transport has been studied predominantly in the context of ischemic stroke. In one study, liposomes were intravenously administered at time points of 0.5 h, 4 h, 24 h, and 48 h in a mouse model of stroke induced by transient middle cerebral artery occlusion [27]. Results indicated that selective liposomal accumulation in the mouse brain correlated with biphasic BBB breakdown in stroke regarding both time and distribution, preceded neuronal cell death, and persisted 24 h after injection. More specifically, enhanced transcellular transport caused increased liposomal accumulation at time points of 0.5 h, reduction in transport vesicles caused decreased liposomal accumulation between 4 and 24 h, and the enhancement of both transcellular and paracellular pathways caused increased liposomal accumulation 48 h after stroke. Moreover, the uptake of liposomes by glial cells increased 2–3 days after stoke, suggesting the potential role of liposomes to inhibit post-stroke inflammatory responses and promote repair. These results provide evidence for the neuroprotective effect of liposomes during two therapeutic windows [27].

In another study, PEGylated liposomes were conjugated with HAIPRH (T7), a peptide targeted to transferrin receptors, and loaded with ZL006 [5, 3-dichloro-2-hydroxybenzylamino)-2-hydroxybenzoic acid], an uncoupling agent of ischemia-induced reactions [26]. To measure the biodistribution of ZL006, coumarin-6-labeled liposomes were intravenously administered to Sprague-Dawley rats; after sacrifice, the rat brain was perfused and examined for fluorescence. Results indicate that coumarin-6-labeled T7-PEGylated liposomes distributed extensively throughout the brain region. Moreover, the penetration of T7-PEGylated liposomes was greater than that of PEGylated liposomes minus T7. In brain capillary endothelial cells, the uptake of T7-PEGylated liposomes was concentration dependent, yet still significantly higher than the uptake of PEGylated minus T-7. These results provide evidence of transferring receptor mediated endocytosis [26], demonstrating the duality of liposomes for utilizing both structural and chemical mechanisms.

#### 3.1.3. Focused Ultrasound-Enhanced Drug Delivery

In contrast to nanoparticle-assisted drug delivery and selective liposomal transport, which mask the chemical structure of drugs and thereby facilitate BBB crossing, focused ultrasound-enhanced delivery alters structural features of the BBB itself. More specifically, low-intensity focused ultrasound induces the oscillation of intravenously administered microbubbles, subsequently resulting in “acoustic cavitation” and the temporary opening of capillary tight junctions and the BBB [39]. In theory, such induced BBB opening may allow previously impermeable drug therapeutics to now cross the BBB and reach target brain tissue.

Focused ultrasound-enhanced drug delivery has been investigated for the treatment of various CNS disorders. In one recent study, human patients with early Alzheimer’s disorder underwent magnetic resonance-guided low-intensity focused ultrasound treatment of the hippocampus and entorhinal cortex (220 kHz) and microbubble injection, followed by magnetic resonance imaging with gadobutrol IV to evaluate BBB properties [39]. The study reported that treatments caused the immediate parenchymal enhancement of approximately 95 ± 4% of the targeted volume, indicating an enhanced BBB permeability of approximately 29% (14 to 71%) of the overall hippocampus volume. Moreover, this enhancement resolved within 24 h, and neither off-target parenchymal enhancement nor post-treatment neurological changes were observed [39].

In addition to applications in Alzheimer’s disease, magnetic resonance-guided focused ultrasound with the intravenous injection of microbubbles has also been administered to patients with confirmed or suspected high-grade glioma in combination with systemically administered chemotherapy [104]. T1-weighted magnetic resonance imaging revealed an immediate contrast enhancement of 15–50%, which resolved approximately 20 h later. For the few patients with available peritumor results, it was noted that the concentration of chemotherapy in sonicated tissue, in which BBB was disrupted, was higher than the concentration of chemotherapy in unsonicated tissue, in which the BBB was undisrupted [104].

In another study, dextran was intranasally administered to experimental C57BL/6 mice, followed by focused ultrasound on the left caudate putamen in combination with systemically administered microbubbles [28]. After treatment, dextran delivery outcomes were evaluated with fluorescence imaging. Results from this study indicate the focused-ultrasound technique enhanced the intransal delivery of dextran of the left caudate putamen in comparison to the right caudate putamen, which received intranasal dextran minus focused ultrasound. The results were significant, corresponding to an 8-fold increase in fluorescent intensity with focused ultrasound [28] but has yet to be validated in humans. Although there have been some positive results for the use of focused ultrasound-enhanced drug delivery, even the temporary opening of the BBB can be a potential issue, so methods that utilize endogenous properties could be better at minimizing damage.

#### 3.1.4. Exosomes

Exosomes are crucial for intracellular communication in the body, but they can also be used to deliver drugs and therapeutic molecules across the BBB to the brain. They are nano-sized vesicles that are produced by almost all types of cells [32]. Exosomes are ideal for the treatment of CNS disorders because they are small in size and are stable [32]. Because of these favorable properties, mesenchymal stem cell (MSC)-derived exosomes have recently been investigated for the treatment of multiple CNS disorders, including epilepsy, stroke, brain cancer, and TBI.

In a recent study conducted with a pilocarpine mouse model of epilepsy, anti-inflammatory MSC-derived A1-exosomes were administered intranasally to mice after pilocarpine-induced status epilepticus to attempt the prevention of neuronal and functional damage caused by this epileptic episode [105]. The authors report that the exosome treatment in mice resulted in reduced neuroinflammation, decreased neuronal loss, and the prevention of abnormal neurogenesis and the loss of cognitive function [105]. These findings are exciting in the scope of epilepsy treatment, but the utilization of exosomes in the treatment of epilepsy patients for the delivery of either endogenous anti-inflammatory molecules or of medication still needs to be investigated much further.

Stem cell-derived exosome treatment has also been tested in animal models of TBI and stroke. After treatment with stem cell-derived exosomes post-stroke, young mice showed a decrease in lesion volume on MRI and improved motor function [106]. In addition to exosome treatment in animal models of stroke, there is an ongoing phase 2 clinical trial (NCT03384433) for MSC-derived exosome treatment in patients with acute ischemic stroke to help promote neurogenesis. No results have been posted as of now for this clinical trial, but the use of this treatment in stroke patients seems hopeful. Similar to stroke, exosome treatment for TBI has been tested in animal models of the disease. After intravenous infusion of MSC-derived CD63+CD81+ exosomes shortly after injury, mice with TBI showed improvements in cognitive damage [33]. Although these results seem promising, human studies are needed to assure that the results observed in animal models of the disease can be translated to a heterogeneous population of patients with TBI.

MSC-derived exosomes for the treatment of brain cancer have also been investigated recently, due to the shared therapeutic challenge of conquering the BBB. One study cultured MSC transduced with CXCR4+TRAIL to recognize the tumor (CXCR4) and to selectively induce apoptosis (TRAIL) in an MDA-MB-231Br SCID mouse model [107]. The CXCR4+TRAIL exosomes administered alongside carboplatin, a chemotherapy drug, by carotid injection every other day for 14 days decreased the tumor size compared to the group that was given a combination of carboplatin and exosomes that did not contain CXCR4+TRAIL [107]. These results show that this particular combination therapy of CXCR4+TRAIL exosomes and carboplatin improved the therapeutic outcome in this model. Although, this type of treatment in humans with brain cancer has yet to be investigated.

### 3.2. Chemical Challenges

#### 3.2.1. Receptor-Mediated Transcytosis

In order to successfully deliver pharmaceutical compounds across the BBB, the chemical properties of the endothelial cells that partially comprise the BBB must be overcome. One drug delivery method by which the chemical properties, such as enzymatic degradation, can potentially be avoided is receptor-mediated transcytosis (RMT). The RMT of drugs occurs when the drug is recognized as a ligand by a receptor on the luminal side of the BBB endothelial cells. The drug-bound receptor then is brought into the cell via endocytosis, and this exosome travels to the abluminal side of the endothelial cell where it fuses with the plasma membrane to release the drug-receptor conjugate [108,109,110,111]. Due to its utilization of an innate transport system that does not involve any inflicted damage to the BBB and its ability to avoid the chemical properties of endothelial cells, RMT has been implemented in the treatment of various CNS disorders.

There are multiple receptors on the surface of BBB endothelial cells that have been targeted for the delivery of CNS-disorder medications, including the transferrin receptor (ischemic stroke, glioblastoma) and insulin receptor (Alzheimer’s disorder) [112,113,114,115,116]. Recently, a mouse anti-transferrin receptor nanobody was discovered that is able to cross the BBB via RMT and deliver a bioactive peptide to the mouse brain in vivo. These nanobodies were administered to the mice peripherally (by either intraperitoneal, intravenous, or subcutaneous injections) [115]. This is just one study that shows that RMT is a plausible drug delivery method to circumvent the chemical barrier of the BBB. In addition to this mouse study, clinical trials for RMT drug delivery via the transferrin receptor [117] and insulin receptor [118] are ongoing to counteract neurodegeneration in a rare genetic disease. RMT is a promising method to deliver therapeutics through the BBB to the diseased brain that is likely to be implemented even more in the near future.

#### 3.2.2. Receptor Agonists or Antagonists and Enzyme Modulation

The BBB contains nuclear receptors that influence the expression of drug-metabolizing enzymes, like CYP enzymes [60,61]. These receptors have also been found to be upregulated in certain CNS disorders, such as drug-resistant epilepsy, corresponding to increased CYP enzymes and decreased drug penetration across the BBB [61,62]. By modulating these receptors, the chemical barrier of the BBB can be minimized and drug availability can be increased. Multiple nuclear receptors in the BBB control the expression of various CYP enzymes. For example, the constitutive androstane receptor (CAR) and pregnane X receptor (PXR) have been found to bind to *CYP2C* gene promoters in the human BBB [119]. Additionally, silencing the glucocorticoid receptor (GR), another nuclear receptor found in the BBB, in vitro in human epileptic brain microvascular endothelial cells was found to decrease protein levels of CYP3A4 while increasing the penetration of phenytoin across a dynamic in vitro BBB system [61]. The dysregulation of GR has been implicated in other CNS disorders in addition to epilepsy, including Alzheimer’s disorder [120]. GR antagonists, such as mifepristone and ketoconazole [121], have been looked into as potential treatments or co-administered therapies for these diseases, including clinical trials sponsored by Bristol-Myers Squibb (NCT00860275) and Pfizer (NCT00931073) to determine the safety and efficacy of supplementing treatments for Alzheimer’s disease with ketoconazole (NCT00860275, NCT00931073). Mifepristone treatment in a mouse model of Alzheimer’s disease was also found to decrease and prevent amyloid-β levels in the CNS [122], demonstrating the potentially beneficial effects of GR antagonism. Although modulating these receptors seems to be a promising therapy for the management of certain CNS disorders, there could be risk involved in targeting these receptors due to the fact that GR and other nuclear receptors are not only expressed in the brain endothelium but rather expressed ubiquitously across the human body.

### 3.3. Transport-Mediated Challenges

#### Carrier-Mediated Transport and Analogs of Solute Carrier Proteins

The luminal and abluminal plasma membranes of the endothelial cells that partially make up the BBB are home to various influx and efflux transporters that help to protect the brain, only letting in particular molecules and getting rid of others by effluxing them back into the blood [123]. Although this property of the BBB guards the brain from toxins and other harmful compounds, it poses a challenge for delivering molecules to the brain that are meant to get through, such as pharmaceuticals. Carrier-mediated drug transport utilizes the innate transport systems at the BBB to obtain an improved yield of the drug across the BBB and into the brain [123]. Influx transporters can be targets for drug delivery. Hydrophilic molecules, such as nutrients and other essential molecules, are transported into the brain across the BBB via various solute carrier proteins and transporters. Some of these transporters include GLUT1 (D-glucose), MCT1 (L-lactate), CRT (creatine), and CNT2 (nucleosides) [110,123]. Developing drugs that have a similar structure to the molecules that are recognized by these transporters can be an opportunity to improve drug transport across the BBB. Gabapentin, an anticonvulsant medication often used to treat epilepsy patients, and melphalan, a medication used to treat brain cancer, are transported into the brain via carrier-mediated transport through the LAT1 (SLC7A5) transporter [123,124,125]. The LAT1 transporter is a large neutral amino acid influx transporter in the BBB [124,125], but because the structures of these compounds are similar enough to that of the innate substrate for LAT1, these drugs are recognized by this transporter and influxed into the endothelial cells via active transport [126]. Although, the expression of LAT1 and other transporters can be altered with disease, which is something that should be considered for the development of any therapy that will enter the brain through carrier-mediated transport [126].

Just as drugs can be influxed via carrier-mediated transport, they can also be substrates of BBB efflux transporters, such as Pgp and other ATP-binding cassette transporters, OAT3, OATP2, etc. Drugs that are efflux transporter substrates are less likely to have high brain bioavailability. Although, developing methods to bypass or avoid the efflux transporter system of the BBB for improved drug delivery to the brain is a continued effort.

A recent study using male Wistar rats investigated the effects of intranasal Pgp-substrate drug delivery in combination with either the intranasal or intravenous administration of the Pgp inhibitor PSC-833. Interestingly, this group found that intranasal drug administration combined with intravenous PSC-833 administration increased drug delivery to the brain, whereas the combined intranasal delivery of both drugs did not improve drug delivery to the brain, measured by in vivo dual probe microdialysis [127]. In animals that did not receive the Pgp inhibitor, the absorption of the intranasal drug was minimal but could be increased by the systemic administration of PSC-388 [127]. These results show that BBB efflux transporters can affect the drug delivery even of medications that are administered intranasally. The fact that the systemic administration of the Pgp inhibitor showed an effect but not intranasal administration demonstrates that although intranasal drug administration is believed to circumvent the BBB, BBB efflux transporters can still be a hurdle to overcome for drug delivery to the brain.

Pgp upregulation has been well recognized as a major problem for drug delivery through the BBB in patients with drug-resistant epilepsy and brain cancer [128,129,130,131,132]. Pgp inhibition has showed promising results in increasing drug delivery to the brain in drug-resistant brain cancer in mice [133,134], and in the human aspect, there was a clinical trial (NCT00011414) that was completed in 2019 where tariquidar was co-administered with one of three anti-cancer drugs in patients. Although there has been no published data yet from this clinical trial, it is worth mentioning that Pgp inhibition is on the way to being put into effect in the treatment of some human CNS disorders.

The endogenous transporter systems, both influx and efflux, can have a major effect on the amount of drug that is able to pass through the BBB and into the brain. Counteracting BBB drug efflux transporters, such as Pgp, remains an unresolved issue for drug delivery to the brain through the BBB and warrants further investigation.

## 4. Key Factors to Be Considered for Enhancing Drug Delivery across the BBB

### 4.1. Age

Age has been an important factor for consideration for the treatment of disease, especially to facilitate safe but adequate drug delivery across the BBB to the aging brain. Pathophysiological changes that occur with aging could contribute to target sensitivity that may affect poor drug responsiveness [135,136]. The contributions of aging to cellular damage are interrelated and include oxidative stress, epigenetic changes, genomic instability, telomere attrition, the dysregulation of cell signaling and inflammatory responses, and senescence. Cellular senescence includes a senescence-associated secretory phenotype and biomarkers such as β-galactosidase where transcriptomic studies have observed age-associated increases in the number of brain endothelial cells that exhibit high levels of senescence-related gene expression [137]. One regulator of senescence that mediates BBB dysfunction is sirtuin-1, and loss of sirtuin-1 with aging is associated with BBB dysfunction [138].

The evidence thus far has also demonstrated a potential influence of aging on BBB properties, including permeability. For example, inflammation and scar formation-associated genes were found to be upregulated in astrocytes of aged Sprague-Dawley rats, whereas the level of brain glucose uptake and the associated glucose transporter, GLUT1, expression was decreased in astrocytes from adult and aged Wistar rats aged in vitro [139,140]. Additionally, pericyte expression was found to decrease via deficient PDGFRβ signaling in adult and aging mouse brains, corresponding with overall BBB dysfunction [141].

Pgp may also contribute to differential drug delivery between age groups. For example, aging was associated with decreased Pgp function in human males as measured by positron emission tomography of the volume distribution of (R)-[(11)C]verapamil [142]. As Pgp functions to pump drugs from the brain back into the blood circulation, the decreased expression of this transport protein supports the hypothesis that aging is associated with increased BBB permeability, thereby facilitating drug delivery and in some instances, possibly disturbing the therapeutic index in the brains of elderly patients.

Conversely, in another study, adult rats, neonatal rats, and hypoxic-ischemic neonatal rats were injected with ^125^I-labeled β-nerve growth factor (β-NGF) through peripheral administration. Based on radioactivity assessments, BBB permeability to β-NGF was found to be higher in the neonatal groups compared to the adult group. These results suggest that aging could compromise BBB integrity associated with drug penetration, information that evidently conflicts with the previous studies discussed. However, it is also possible that the BBB of the newborn rats demonstrated increased permeability to β-NGF due to the incomplete development of the BBB, or other molecular factors could play a role during development. Further investigation is necessary to distinguish the underlying cause of these alterations, which are age-dependent [143].

There are controversies about how aging affects the BBB properties, where aging seems to demonstrate some effect on target sensitivity, compromising drug passage and accumulation in the brain with poor clearance [17,144]. Therefore, such variability and demographic factors must be considered in drug delivery treatments and pre-clinical research, to sustain drug efficacy and minimize adverse consequences.

### 4.2. Biological Sex

In the “Age” section, it was noted that Pgp function as measured by positron emission tomography of the volume distribution of (R)-[(11)C]verapamil decreased with age in human males. However, these results were not observed among female subjects, possibly providing insight into the distinct mechanisms of aging within human males and females. Beyond within-sex distinctions, variability in Pgp function has also been documented between biological sexes. In particular, the volume of distribution of the drug was higher among young females in comparison to their young male counterparts, suggesting decreased Pgp function in young females. Potential explanations for these findings include inter-individual differences or the action of female reproductive hormones estrogen and progesterone on Pgp function [142,145].

Additionally, biological sex may influence drug pharmacokinetic properties. In a recent study, BBB penetration and the clearance of letrozole, a hormone-based chemotherapeutic agent, were examined in male and female Sprague-Dawley rats. More specifically, the rate of letrozole clearance was significantly slower in female rats in comparison to male rats, corresponding to a half-life of 34 h and 9 h, respectively. As a result, female rats showed higher drug concentrations in both plasma and brain samples. Despite these differences, T_max_, the time for maximum drug concentration, was comparable between male and female rats, suggesting that the observed differences involved elimination pathways, not absorption pathways [146]. An additional discussion of drug pharmacokinetics is located in the “Pharmacokinetic and Pharmacodynamic Properties” subsection.

### 4.3. Route of Administration

The route of drug administration is an important factor modulating bioavailability to the brain, and several approaches have been recently tested to circumvent the BBB. The delivery paths to the brain include the oral, rectal, intravenous, intramuscular, buccal, sublingual, intranasal, and intrathecal routes [147,148]. The distinct routes of administration for drugs utilized in the treatment of selected CNS disorders are listed in Table 2.

Drugs administered through the oral route undergo first-pass metabolism through the liver upon entering the systemic circulatory system. Drugs administered through the rectal, intravenous, intramuscular, buccal, and sublingual routes enter directly into the systemic circulatory system. Upon circulation, these drugs must cross the BBB in order to reach the target brain tissue. As referenced earlier, structural and functional properties of the BBB present multiple hurdles to pharmaceutical compounds, thereby decreasing the concentration of drugs at the target brain tissue [200]. However, drugs administered through the intranasal and intrathecal routes bypass the BBB, avoiding the observed reduction in drug bioavailability, altogether [201].

More specifically, the intranasal route involves drug administration via the nasal cavity. Through the intracellular pathway, drugs are first absorbed by olfactory sensory neurons and pass through olfactory unsheathing cells and the olfactory bulb to eventually reach the CNS. Through the extracellular pathway, drugs cross tight junctions of support cells in the nasal mucosa and travel to the subarachnoid space for transport to distal CNS regions [202]. Recent studies have demonstrated potentially promising results of intranasal drug administration. In particular, albumin, a drug carrier protein unable to naturally cross the BBB, was found to be distributed to all regions of the CD1 mouse brain within five minutes of intranasal administration [203]. Additionally, the concentration of dantrolene, a neuroprotective agent for neurodegenerative disorders, was found to be sustained even 180 min after intranasal administration in C57BL/6 mice. This duration was significantly longer than the duration associated with the oral administration of this drug, in which the concentration of dantrolene was depleted within 120 min [204].

Alternatively, through an intrathecal route, drugs are administered via the spinal canal directly into the cerebrospinal fluid. In theory, drugs travel with the cerebrospinal fluid for later absorption at the arachnoid villi and ventricular walls of the brain and the lymphatic system [205]. However, this route was historically overlooked, as it was suggested that the diffusion of drugs into the brain parenchyma was significantly slower than the elimination of drugs from the cerebrospinal fluid compartment via convection and bulk flow. As a result, the majority of drugs return to the peripheral blood circulation, where they would then have to cross the BBB for CNS entry. Due to this rapid elimination, penetration into the brain was considered limited [206]. More recently, however, the intrathecal route has been reevaluated to be a potentially beneficial technique for the administration of protein therapeutics [148].

In summary, the utilization of different routes of drug administration (Table 2) may provide opportunities to improve the bioavailability and duration of drug treatment in the CNS. However, as a result of improved drug efficacy, medical practitioners must also appropriately decrease the drug dosage to prevent drug interactions, toxicity and other potentially adverse effects among individuals with CNS disorders [207].

### 4.4. Pharmacokinetic and Pharmacodynamic Properties

In simple terminology, pharmacokinetics describes the body’s action upon the drug, including absorption, distribution, metabolism, and excretion [208]. The first stage, absorption, is the process from drug administration to systemic circulation. As discussed previously, the route of administration affects the bioavailability and duration of drug treatment in the brain. Between administration and absorption, however, drugs must first be released from their original pharmaceutical form. The duration of this process varies between drugs, from fast-acting to extended-release compounds, causing differential onsets of action and side effects. Drug distribution is determined by both the drug’s biochemical properties, such as size, polarity, and binding properties, and patient physiology, including fluid status and protein concentration. Regarding the CNS, a number of anti-seizure medications (e.g., oxcarbazepine, phenytoin, valproate, and zonisamide) and anti-stroke medications (e.g., aspirin and rivaroxaban) are moderately to highly bound to plasma serum proteins (see Table 2). As only a free drug is able to act on target brain tissue, an increased concentration of the plasma proteins albumin and α-acid glycoprotein may decrease the concentration of pharmacologically active drugs in the body [209]. The half-life of the drug, i.e., the time required for 50% of the initial concentration to break down, also influences the final yield and concentration within the brain [208]. Compounds with a short half-life are eliminated more quickly than those with a longer half-life. Aspirin, an antiplatelet agent, for example has a significantly shorter half-life of 15–20 min (see Table 2), suggesting a short duration in the CNS and the dosage adjustment is accordingly tailored for optimum efficacy.

Conversely, pharmacodynamics is the drug’s action upon the body and its effects on receptor binding and downstream chemical interactions. Pharmaceutical drugs may exhibit either a direct effect, in which drugs interact with central receptors or enzymes that generate a biochemical effect, or an indirect effect, in which drugs interact with proteins located upstream from the central pathway [210]. Direct versus indirect action becomes relevant to drug delivery, as these mechanisms directly influence the onset of drug action via immediate or delayed effects [211]. Additionally, chronic exposure to certain drugs may cause the upregulation or downregulation of associated receptors and changes in receptor sensitivity, resulting in decreased efficacy with prolonged treatment [210]. Regarding the CNS, such pharmacodynamic tolerance has been reported in both epilepsy and brain cancer [212,213]. For instance, in epilepsy, tolerance to benzodiazepines, a class of anti-seizure medications that inhibit the neurotransmitter GABA chief inhibitory receptor, may result from decreased allosteric interactions between benzodiazepine recognition sites and the GABA receptor or reduction in binding sites. Along with benzodiazepines, evidence of tolerance has been demonstrated in animal models administered with the first-generation anti-seizure medications carbamazepine, phenobarbital, primidone, and valproate along with second and third-generation anti-seizure medications gabapentin, lamotrigine, levetiracetam, pregabalin, topiramate, vigabatrin, and zonisamide [212]. In brain cancer, cancer cells may downregulate chemotherapeutic target gene expression, thereby reducing efficacy. Other non-pharmacodynamic mechanisms of anti-cancer drug resistance include the alteration of drug pumps, detoxification mechanisms, reduced apoptosis, altered proliferation, and increased DNA damage repair [213].

The pharmacokinetic and pharmacodynamic phenomena of drugs are particularly complicated in the presence of comorbidities [214,215,216]. Patients with comorbidities may be administered treatment to control multiple conditions, where potential interactions between drugs is inevitable and may alter the function or decrease the efficacy of the other. In these situations, the pharmacokinetic and pharmacodynamic properties of each drug must be carefully examined prior to co-administration.

## 5. Concluding Remarks and Future Research

In the past decades, drug delivery challenges in brain disorders have been increasingly recognized and investigated. Significant progress has been made thus far, as demonstrated by the multitude of novel drug delivery methods beyond the standard care for epilepsy, stroke, brain cancer, traumatic brain injury and other CNS disorders.

Despite this rapid progress, barriers to drug delivery are often inherent in the pathophysiology of each CNS disorder and, therefore, may be difficult to eradicate completely without potentially further disturbing CNS homeostasis or alternate functions. As a result, drug delivery challenges in brain disorders, especially those involving structural and functional properties of the BBB alteration, remain an active area of research among the scientific and medical community. As future research proceeds, a consideration of clinical and biological variables, including age, biological sex, the route of drug administration, and the pharmacokinetic and pharmacodynamic properties of drugs are integral to further improving drug delivery to the brain.

## Figures and Tables

**Table 1:** Featured novel drug delivery methods in central nervous system disorders in human and animal models.

CNS Disorders	Novel Drug Delivery Methods	Description	References
Epilepsy	Electrophoretic drug delivery	The microfluidic ion pump detects seizure activity and electrophoretically pumps ions across the ion exchange membrane to deliver the localized treatment of inhibitory neurotransmitters, tested in mice.	[19,20]
	Implanted intracerebroventricular delivery system	The system (clinicaltrials.gov identifier NCT02899611) pumps the anti- seizure medication valproic acid into cerebrospinal fluid for long-term treatment in epilepsy patients.	[21]
	Microencapsulation of anti-seizure medications	Polymer cores loaded with the anti-seizure medication lacosamide are covered with drug-free polymer shells, tested in vitro using artificial cerebrospinal fluid.	[22]
	Nanoparticles	Glucose-coated gold nanoparticles are conjugated with the anti-seizure medication lacosamide for intravenous administration in rats.	[23]
		Chitosan–lecithin nanoparticles were loaded with phenytoin for intranasal administration in mice.	[24]
Stroke	Macrophage migration inhibitory factor antagonist ISO-1	Intravenous administration of ISO-1 (4,5-Dihydro-3-(4-hydroxyphenyl)-5-isoxazoleacetic acid methyl ester) following middle cerebral artery occlusion in vivo in rats.	[25]
	Liposomes	T7-conjugated PEGylated liposomes were loaded with neuroprotectant and nNOS/PSD-95 inhibitor ZL006 in vivo in rat and mouse models of stroke.	[26,27]
	Focused ultrasound-enhancedintranasal delivery	Intranasal administration of dextran in vivo in mice was followed by focused ultrasound and systemic administration of microbubbles.	[28]
Brain Cancer	Bioresorbable electronic patch	Patch performs long-term drug release and mild-thermic actuation increases drug permeation in a mouse model of brain tumor.	[29]
	Nanoparticles	Cornell prime dots with α_v_integrin-binding/nontargeting peptides and PET labels delivered anti-cancer drug dasatinibin in a mouse model of glioblastoma.	[30]
Traumatic Brain Injury (TBI)	Exosomes	Exosomes derived from mesenchymal stem cells (MSC) containing biologically active molecules that aid in reducing inflammation in TBI; intravenous delivery; can cross the blood-brain barrier, shown in animal models.	[31,32,33]
	Nanoparticles	Poly(lactic-co-glycolic acid) nanoparticles in vivo in mice to deliver siRNA for the treatment of TBI; polysorbate 80-coated nanoparticles for receptor-mediated transport via lipoprotein receptor.	[34,35]
Other CNS Disorders	Supramolecular del (Parkinson’s disease)	Hydrogel loaded with amino acid L-DOPA rapidly releases drug after intranasal delivery in mice.	[36]
	Nanoparticles (Parkinson’s disease)	Protocells were co-loaded with Parkinson’s disease drugs levodopa and curcumin and lipid bilayer was modified for brain targeting via intraperitoneal injection in a mouse model of Parkinson’s.	[37]
	Oral and maxillofacial device (Parkinson’s disease)	Device implanted in the oral or maxillofacial region delivers drug to brain via the respiratory mucosa in an in vivo rabbit model.	[38]
	Magnetic resonance-guided low-intensity focused ultrasound (Alzheimer’s disease)	Magnetic resonance-guided low-intensity focused ultrasound treatment of the hippocampus and entorhinal cortex reversibly opens a large area of blood-brain barrier in humans.	[39]

**Table 2:** Properties and routes of administration of selected drugs for epilepsy, stroke, cancer and traumatic brain injury.

Drug Name and Classification	Properties	Route of Administration	Potential Challenges	References
Anti-seizure medications				
Carbamazepine	Prolonged T_max_ (6–12 h), insoluble, plasma half-life 35 h	Oral, rectal	Rectal administration caused irritating and cathartic effects, CYP3A4induction	[149,150]
Gabapentin	Soluble, plasma half-life 5–7 h	Oral	Potential drug interactions	[151,152,153]
Lamotrigine	Plasma half-life 29 h	Oral, rectal	Substrate of P-glycoprotein	[154]
Levetiracetam	High bioavailability, plasma half-life 6–8 h	Oral, intravascular, intramuscular, rectal	Substrate of P-glycoprotein	[154,155,156]
Oxcarbazepine	Rapidly reduced to active metabolite 10,11-dihydro-10-hydroxy-carbamazepine, plasma half-life 1–3.7 h; plasma half-life of monohydroxy derivative ~9.3 h	Oral	~40% bound to plasma protein, CYP3A4 induction	[150,157]
Phenobarbital	Poor water solubility, plasma half-life 100 h	Oral, intravascular, rectal	Substrate of P-glycoprotein, CYP450 inducer	[154,158]
Phenytoin	Poor water solubility, plasma half-life 22 h	Oral, intravascular	Highly bound to serum proteins, substrate of P-glycoprotein	[154,159]
Valproate	Fatty acid derivative, plasma half-life 4–16 h	Oral, intravascular, rectal	Highly bound to serum proteins	[159]
Zonisamide	Plasma half-life 50–69 h (plasma)/105 h (RBCs)	Oral	~40% bound to plasma proteins	[160]
Stroke medications				
Atorvastatin (statin)	Highly soluble, plasma half-life 7 h	Oral	Extensive first-pass metabolism, low oral bioavailability (14%), substrate of P-glycoprotein	[161]
Apixaban (anticoagulant)	Rapidly absorbed, plasma half-life 12 h	Oral	Substrate of P-glycoprotein and breastcancer resistance protein	[162,163]
Aspirin (antiplatelet)	Polar, small, plasma half-life of 15–20 min, platelet-inhibitory effect until platelet death (~10 days)	Oral, rectal, intravenous	Highly bound to albumin (87% in vitro, ~93% in vivo)	[164,165]
Clopidogrel (antiplatelet)	Inactive prodrug, 2-step bioactivation process (esterase hydrolysis with CYP2C19 and CYP3A4), plasma half-life 6 h	Oral	Substrate of P-glycoprotein, low BBB permeability (50%), only ~15% is activated by CYP enzymes	[166,167,168]
Dabigatran (anticoagulant)	High polarity, low bioavailability (dabigatran not bioavailable but bioavailability of prodrug abigatranetexilate increases slightly to is 3–7%), plasma half-life 8 h	Oral	Prodrug is substrate of P-glycoprotein	[166,169]
Hydrochlorothiazide (antihypertensive)	Very poor water solubility, not metabolized, plasma half-life 6–12 h	Oral	Low absorption rate	[170,171]
Rivaroxaban (anticoagulant)	CYP3A4/3A5 and CYP2J2 metabolism, high bioavailability (~80–100% with 10 mg dose), plasma half-life 5–9 h,	Oral	Substrate of P-glycoprotein and breast cancer resistance protein, high plasma protein binding ~92–95%	[166,169,172]
Tissue plasminogen activator	Crosses BBB via LDL receptor-related protein-mediated transcytosis, inhibits P-glycoprotein, plasma half-life 4–5 min	Intravenous	Short window of administration (maximum 3–4.5 h), risk of brain hemorrhage	[173,174,175]
Warfarin (anticoagulant)	Rapid absorption, CYP2C9 metabolism, plasma half-life 20–60 h	Oral, intravenous	Substrate of P-glycoprotein	[166,176,177]
Cancer drugs				
Carmustine	Lipophilic, rapidly crosses BBB, plasma half-life 15–30 min	Intravenous	Rapidly Metabolized	[178]
Doxorubicin	Large size (greater than 0.4 kDa), plasma half-life 48 h	Intravenous	Too large for diffusion through phospholipid bilayer or intraendothelial cell junction pores, does not cross blood-brain barrier	[179,180]
Everolimus	Large size (MW ≈ 1000), CYP3A4, CYP3A5 and CYP2C8 metabolism, plasma half-life 28 h	Oral	Substrate of P-glycoprotein, variable oral bioavailability	[181,182]
Lomustine	Lipophilic, rapidly crosses BBB, initial plasma half-life 6 h, second phase plasma half-life 1–2 days	Oral	Rapidly metabolized	[178,183]
Temozolomide	Small size, lipophilic, prodrug, plasma half-life 1.8 h	Intravenous	Resistance to temozolomide among 50% of patients with glioblastoma multiforme	[184,185]
TBI treatments				
Phenytoin	Free (unbound) drug can cross BBB, plasma half-life 22 h	Oral, Intravenous	90% bound to serum albumin, 95% metabolized by liver, can cause dizziness in patients, careful dosing regimen must be followed due to metabolic enzyme saturation	[186,187,188]
Levetiracetam	Serum half-life 6–8 h	Oral, Intravenous	Substrate of P-glycoprotein	[186]
Hypertonic saline	Contains a higher concentration of NaCl than the plasma and interstitial fluid	Intravenous	Caution must be taken with patients who have congestive heart failure or renal insufficiency	[189,190,191]
Mannitol	Contains a higher concentration of mannitol than the plasma and interstitial fluid	Intravenous	Unwanted blood-brain barrier damage can occur with high levels of hyperosmolar mannitol, eliminated quickly through renal excretion	[190,192,193]
Docosahexaenoic Acid (DHA)	Omega-3 polyunsaturated fatty acid, passive diffusion across BBB, plasma half-life 48 h for repeated administration	Oral	Partially metabolized by CYP enzymes	Phase 2 clinical trial (NCT03345550) completed in July 2021 [194,195]
Propranolol (beta-blocker)	Lipophilic, plasma half-life 3–6 h	Oral, intravenous	Mostly eliminated through renal excretion	[196,197,198]
Mesenchymal stem cells (only validated in animal models thus far)	Too large to cross BBB, release exosomes that can cross BBB	Intra-arterial, intravenous, intracerebral	Are not able to cross the BBB but act on brain inflammation from the periphery	[32,199]
